# Polymeric Inserts Containing Eudragit^®^ L100 Nanoparticle for Improved Ocular Delivery of Azithromycin

**DOI:** 10.3390/biomedicines8110466

**Published:** 2020-10-31

**Authors:** Shiva Taghe, Shahla Mirzaeei, Raid G. Alany, Ali Nokhodchi

**Affiliations:** 1 Pharmaceutical Sciences Research Center, Health Institute, Kermanshah University of Medical Sciences, Kermanshah 6714415153, Iran; shivataghe@gmail.com; 2 Nano Drug Delivery Research Center, Health Technology Institute, Kermanshah University of Medical Sciences, Kermanshah 6714415153, Iran; 3 Drug Discovery, Delivery and Patient Care (DDDPC), School of Life Sciences, Pharmacy and Chemistry, Kingston University, London KT1 2EE, UK; R.Alany@kingston.ac.uk; 4 School of Pharmacy, The University of Auckland, Auckland 1023, New Zealand; 5 Pharmaceutics Research Laboratory, School of Life Sciences, University of Sussex, Brighton BN1 9QJ, UK

**Keywords:** Azithromycin, controlled drug delivery, Eudragit^®^ L100 nanoparticles, mucoadhesive films, ocular drug delivery

## Abstract

Polymeric inserts containing azithromycin-loaded Eudragit^®^ L100 nanoparticles were developed to sustain the drug release and enhance its ocular performance. The solvent diffusion technique was employed to prepare nanoparticles. The developed nanoparticles (NPs) were fully characterized and investigated. The solvent casting method was used to prepare azithromycin ocular inserts (azithromycin, AZM film) by adding hydroxypropyl methylcellulose (HPMC) or hydroxyethyl cellulose (HEC) solutions after the incorporation of AZM-loaded Eudragit^®^ L100 nanoparticles into plasticized PVA (polyvinyl alcohol) solutions. The optimized nanoparticles had a particle size of 78.06 ± 2.3 nm, zeta potential around −2.45 ± 0.69 mV, polydispersity index around 0.179 ± 0.007, and entrapment efficiency 62.167 ± 0.07%. The prepared inserts exhibited an antibacterial effect on *Staphylococcus aureus* and *Escherichia coli* cultures. The inserts containing AZM-loaded nanoparticles showed a burst release during the initial hours, followed by a sustained drug release pattern. Higher cumulative corneal permeations from AZM films were observed for the optimized formulation compared to the drug solution in the ex-vivo trans-corneal study. In comparison to the AZM solution, the inserts significantly prolonged the release of AZM in rabbit eyes (121 h). The mucoadhesive inserts containing azithromycin-loaded Eudragit^®^ L100 nanoparticles offer a promising approach for the ocular delivery of azithromycin (antibacterial and anti-inflammatory) to treat ocular infections that require a prolonged drug delivery.

## 1. Introduction

The topical administration of antibiotics is the most common route to treat the infections that can affect the anterior segment of the human eye. There are various precorneal dynamic and static barriers that can restrict the delivery of the drug to specific ocular tissues. Besides, rapid elimination of the drug from the target tissues leads to a decreased level of the drug under the therapeutic concentrations. Despite the popularity of the topical dosage forms for ocular drug delivery, they provide low bioavailability (less than 5%) because of the poor precorneal drug retention and penetration. Poor precorneal retention occurs due to factors like rapid tear turnover, blinking reflex, and nasolacrimal drainage, which results in the immediate elimination of the drug after topical administration [1,2]. As a result, frequent instillations of eye drops are needed for maintaining a therapeutic drug concentration on the ocular surface and in the anterior segment of the eyes, which could lead to induced toxicity, dryness, and other side effects. Drug delivery systems that are capable of prolonging the drug contact time with the cornea can enhance the absorption of drugs from the precorneal tissue [3]. AzaSite^®^ (azithromomycin 1.0%) ophthalmic solution was approved in 2007 by the US Food and Drug Administration (FDA) as the first commercially available formulation of ophthalmic azithromycin for the treatment of bacterial conjunctivitis. AzaSite^®^ utilizes a vehicle delivery system called DuraSite^®^, which stabilizes and sustains the release of azithromycin to the ocular surface, leading to a longer drug residence time, less frequent dosing, and an increase in patient compliance. As azithromycin (AZM) is a very poorly water-soluble drug, its formulation is rather difficult, and its aqueous solutions could be prone to recrystallisation on temperature fluctuations. Another ophthalmic formulation is Azyter^®^, which has been approved in Europe, and it contains medium-chain triglycerides, which sometimes cause side effects. Therefore, a safer formulation with less side effects is needed.

Nanoparticles with smaller sizes are suitable for ocular drug delivery because of their low irritation. Additionally, sustained release of the drug from nanoparticles avoids frequent administration of the formulation by patients. On the other hand, like eye drops, nanoparticles also have a short precorneal residence time. Hence, the incorporation of nanoparticles in polymeric inserts with mucoadhesive properties can enhance the precorneal residence time of the drug to overcome the challenge of rapid elimination of nanoparticles from the surface of the cornea [4]. Ocular inserts are sterile solid dosage forms placed into the ocular cul-de-sac, and their shape and size are tailored for ocular drug delivery. They are prepared using different kinds of polymers with or without a loaded drug, which can be utilized for topical administration [5]. The studies have indicated that azithromycin (AZM) is a potent antibiotic against different kinds of bacteria, including Gram-positive, Gram-negative, and atypical bacteria. It also plays a crucial role as an anti-inflammatory agent by avoiding the production of bacterial toxin, which can be harmful to the eye in ocular infection [6,7,8]. One of the important problems related to the topical application of azithromycin formulations is their hydrophobic nature and being barely soluble in water [9]. To achieve an improved topical drug delivery, it is essential to prepare a novel system for AZM delivery. Topical ocular formulations of AZM like inserts containing hydroxypropyl methylcellulose and Eudragit RL100 [10], liposomes [11], nanoemulsions [12], microemulsions [13]), niosomes [14], and poly (lactide-co-glycolide) (PLGA) nanoparticles [15] have been reported. This study reports for the first time on AZM-Eudragit^®^ L100 nanoparticles in a hydroxyethyl cellulose or hydroxypropyl methylcellulose base as an insert to prolong the drug release and prolong the NP residence time.

## 2. Materials and Methods

### 2.1. Materials

Hydroxyethyl cellulose (HEC) and poly (vinyl alcohol) were obtained from Merck (Darmstadt, Germany). Hydroxypropyl methylcellulose (HPMC) was obtained from Sigma–Aldrich Chemical (St. Louis, MO, USA). Eudragit^®^L100 polymer was purchased from Rofarma Italia S.r.l. (Gaggiano, Milan, Italy). Azithromycin powder was purchased from Dr Reddy’s Pharmaceutical Company, Bengaluru, India. Tryptic soy agar (TSA) was purchased from Merck Korea Ltd. (Seoul, Korea).

### 2.2. Preparation of Azithromycin-Eudragit^®^ L100—Nanoparticles (AZM-EUD NPs)

The solvent diffusion technique developed by Fessi et al. [16] was successfully employed to prepare Eudragit^®^ L100 nanoparticles with minor modifications. The technique is based on the deposition of a polymer following the replacement of an organic solvent with water in the system. The method offers a number of advantages, including being simply cost-effective and energy-saving. Eudragit nanospheres were developed utilizing a solvent diffusion nanoprecipitation technique under aseptic conditions. For the preparation of AZM-EUD NPs, amounts of Eudragit^®^ L100 (2.5, 5, and 10 mg/mL) and drug (1 mg/mL) were dissolved in methanol (organic phase). The polymeric solution was added to PVA (72,000 MW, 88% hydrolyzed) aqueous solution (2.5, 5, and 10 mg/mL) under stirring at 100 rpm for 7 h at room temperature for organic solvent removal, where the Eudragit^®^ L100 nanoparticles were formed. The amount of materials used in the aqueous and organic phase was modified to obtain AZM-EUD NPs with an appropriate size, zeta potential, polydispersity index, and drug loading, as shown in Table 1.

### 2.3. Characterization of AZM-EUD Nanoparticles

#### 2.3.1. Particle Size and Zeta Potential Analysis

The particle size distributions and zeta potential of the obtained nanoparticles were determined using Malvern Zeta Sizer 3000 HS (Malvern Instruments Ltd., Malvern, UK). The analysis was performed at room temperature in deionized water as a solvent.

#### 2.3.2. Determination of Entrapment Efficiency of Azithromycin in Nanoparticles

To separate nanoparticles containing AZM from aqueous solutions, the nanoparticle suspensions were subjected to centrifugation (Beckman Coulter Centrifuges, optima-L90k, Indianapolis, IN, USA) for 45 min at 30,000 rpm. Then, the precipitated nanoparticle suspensions were collected and frozen at −70 °C, followed by freeze-drying of the sample (Christ Freeze Dryer ALPHA 2-4, PLUS, 121, Osterode am Harz, Germany). The freeze-dried nanoparticles were kept at 4 °C for further analysis. To determine the azithromycin content, specified amounts of lyophilized AZM-EUD NPs (5 mg) were dissolved in methanol and vortexed. Then, 5 mL of phosphate-buffered saline solution (pH 7.4) was added to the methanolic solution and vortexed for 15 min to extract azithromycin. The amount of drug was analyzed by microbiological assays. Briefly, a calibration curve was obtained by using standard discs containing different concentrations of AZM (0.03–500 µg/mL), which were placed on plates containing *Micrococcus luteus*. The culture medium containing the bacteria and standard discs were incubated at 35 °C for 24 h. The diameter of the inhibition zone surrounding the paper discs was recorded in mm using a vernier caliper, and a standard curve was constructed for the diameter of the inhibition zone (Y) versus log concentration of AZM (X). The obtained equation was Y = 11.032X + 23.38 with an R^2^ value of 0.9869. To determine the amount of AZM in nanoparticles, a certain amount of the methanolic solution containing AZM was placed on a paper disc, and after drying, it was placed on the environment containing *Micrococcus luteus*. The culture medium containing the bacteria and standard discs incubated at 35 °C for 24 h; the concentration of AZM was quantified using the constructed calibration curve. Experiments were performed in triplicate. The entrapment efficiency (EE) was calculated by Equation (1) [17]:

%EE = (actual drug loading)/(theoretical drug loading) × 100 (1)

#### 2.3.3. Fourier-Transform Infrared Spectroscopy (FTIR)

For any molecular interaction between the drug and polymers, the prepared nanoparticles were subjected to FTIR studies (Shimadzu IR PRESTIGE-21, Kyoto, Japan). To this end, 5 mg of dried nanoparticles were mixed with 950 mg of KBr. The mixture was compressed into a disc under 10 tons of pressure for 10 min. FTIR spectra of AZM, Eudragit^®^ L100, PVA, and nanoparticles were obtained between 400–4000 cm^−1^.

#### 2.3.4. In Vitro Cytotoxicity Test

To perform a cell viability test against L929 (mouse fibroblast), a microplate reader was used. Cells (4 × 10^4^ per well) were cultivated and incubated in a 24-well plate for 48 h. One row of the plates that did not receive nanoparticles was considered as the control. AZM-loaded nanoparticles were added to other rows. To all of the wells, 30 µL of MTT (3-(4,5-dimethylthiazol-2-yl)-2,5 diphenyl tetrazolium bromide) and 270 µL of medium containing different concentrations of nanoparticles (125, 250, 500, and 1000 µL/mL) were added and incubated for around 4 h; then, the solutions were removed from each well, leaving the precipitate behind. After removing the solutions, 150 µL of Dimethyl sulfoxide (DMSO) was added to the wells before the plate was examined using the microplate reader. Cell viability was determined by the ratio of absorbance of the test cells and absorbance of the control cells at the wavelength of 570 nm, where the absorbance of the test cells and absorbance of the control cells represent the amount of formazan determined for cells treated with different formulations and for control cells (nontreated), respectively [18].

### 2.4. Preparation of Ocular Inserts

**Figure 1:**
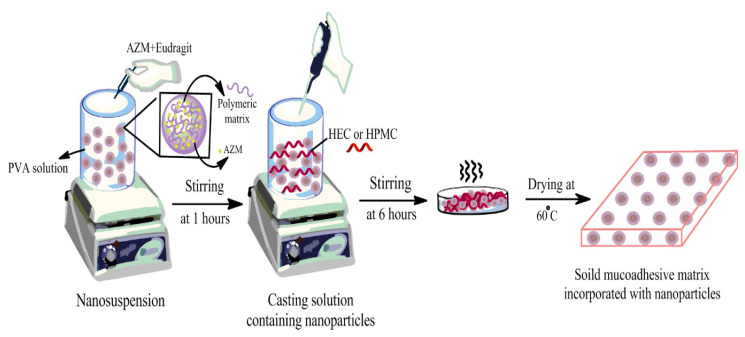
The experimental procedure for the azithromycin (AZM) nanoparticle and solid film preparation.

The HPMC and HEC solutions were obtained by dissolving 1 g of HPMC and HEC in 100 mL of distilled water separately under continuous stirring. AZM films were obtained by an addition of HPMC or HEC solutions in nanoparticle suspension under the stirring conditions for 6 h. The solutions were then poured on an acrylic mold for film casting preparation and were kept on a leveled surface at 60 °C temperature for 12 h to dry the samples [19]. The experimental procedure is shown in Figure 1. After drying, AZM films were taken out and sterilized using UV radiation for 90 min under aseptic conditions and were finally packed in sealed plastic bags stored at room temperature before use. Different formulations of inserts were prepared as shown in Table 2.

### 2.5. Characterization of Prepared Ocular Inserts

#### 2.5.1. Inserts Morphology

The morphology of ocular inserts containing AZM-EUD NPs was examined by SEM (FE-SEM, MIRA3, TESCAN, Brno – Kohoutovice, Czech Republic). For this purpose, a thin layer of gold was deposited on the inserts using a physical vapor deposition method. NPs in films were observed with a scanning electron microscope utilizing secondary electron imaging at 15 kV to investigate the morphology and structure of ocular inserts containing AZM-EUD NPs.

#### 2.5.2. Thickness Measurement

The thickness of the ocular inserts was determined by a digital micrometer (Mitutoyo Manufacturing, Tokyo, Japan) at 10 different positions around the film. The thickness was measured, and the average was taken [20].

#### 2.5.3. Folding Endurance

Folding endurance was measured by frequently folding the inserts at the same place until it broke. The value of folding endurance was determined by counting the number of times the inserts could be folded at the same place without breaking. This test is required to investigate the strength of the inserts to withstand folding. This also indicates the brittleness of the films [20].

#### 2.5.4. Swelling, Moisture Absorption, and Moisture Loss Studies

To measure the swelling percentage of AZM films (*n* = 3), the initial weight of the ocular insert was determined followed by placing the insert in an agar plate (2% *w*/*v* agar in simulated tear fluid, pH 7.4) that was kept at 37 °C ± 1 °C. The prepared AZM films were taken out from the plate after 30 min, and immediately, the water at the surface of the insert was eliminated using filter paper, and AZM films were reweighed. The swelling index was calculated using Equation (2).

To measure the moisture absorption percentage, AZM films were weighed and kept in a desiccator with 79.5% relative humidity (this RH was generated by a saturated solution of aluminum chloride in water). AZM films were removed and reweighed after three days, and the percentage moisture uptake was calculated using Equation (3). To measure the moisture loss percentage, AZM films were weighed and placed in a desiccator containing anhydrous calcium chloride. AZM films were removed and reweighed after 3 days, and the moisture loss percentage was measured utilizing Equation (4) [21].

%Swelling Percentage = (wt. of swollen insert − wt. of initial insert)/(wt. of initial insert) × 100 (2)

%moisture uptake = (Final weight − Initial weight)/(Initial weight) × 100 (3)

%moisture loss = (Initial weight − Final weight)/(Initial weight) × 100 (4)

#### 2.5.5. Mechanical Strength

As inserts need to resist tearing and breaking due to stress created by the blinking of the eyes, therefore, the mechanical strength of the inserts was determined (*n* = 3). To this end, one end of the prepared insert was fixed to a movable clip, while the other end was fixed to the base plate. A thread was tied to the clip and passed over the pulley, to which a small pan was attached to hold weights. A small pointer was attached to the thread that travels over the scale affixed on the base plate. The weights were gradually added to the pan until the insert (that was affixed between two clips) broke. The tensile strength was measured from the ultimate load before separation [20].

#### 2.5.6. Surface pH Determination

To measure the surface pH, the prepared insert was swollen in the plate at 25 °C for 30 min in distilled water. The pH paper was placed on the surface, and after 1 min, the color was evaluated by comparison of the developed colors with the standard color scale [20].

#### 2.5.7. Microbiological Assay Test

The microbiological assays were done on *Micrococcus luteus* (ATCC 4698) by the standard disc diffusion method. Tryptic soy agar (TSA) plates were used to cultivate bacteria. The spread-plate method was used, and the culture medium incubated at 35 °C for 24 h. The samples, including the certain amounts of the drug, were infused into sterile paper discs. The bacteria were suspended in their culture media. Two milliliters (1.5 × l0^8^ colony-forming units (cfu)/mL) of this suspension were applied uniformly on the surface of a nutrient agar plate. Then, sterile paper discs of 6-mm diameters (containing 30 μL of samples), along with standard antibiotic discs, were placed in each plate and incubated at 35 °C for 24 h. The diameter of the inhibition zone surrounding the paper discs was recorded in mm using a vernier caliper. The test was performed, twice with three replicates for each sample [22].

#### 2.5.8. In Vitro Antimicrobial Efficacy and Sterility Testing

Bactericidal effects of AZM films were measured using inhibitory zone measurements against both Gram-positive and Gram-negative organisms, including *Staphylococcus aureus* (ATCC 6538) and *Escherichia coli* (ATCC 35218), respectively [23]. A gelatin pellet infused with the bacterial strain was incubated in 9 mL of tryptic soy broth (Mecrotube^®^; Merck and Co., Seoul, Korea) at 37 °C for 24 h. An aliquot of the suspension of microorganism (0.7 mL) was uniformly spread onto an agar plate (Caso-Agar, Mercoplate^®^; Merck and Co.) and allowed to dry for several minutes. The AZM films (6-mm-diameter discs) were placed on the agar plates and incubated at 37 °C. The sterility test of the ocular inserts (without drug) was carried out for microorganisms, including fungi, the aerobic bacteria, and anaerobic bacteria, utilizing soybean casein digest medium and alternative thioglycolate medium. The positive and negative controls for the observation of growth promotion and sterility were also performed.

#### 2.5.9. In Vitro Release Studies

In vitro release studies were performed using a dialysis tube method. A dialysis bag (molecular weight cut-off 12,000 Daltons, Milan, Italy) was tied at one end, and AZM films and AZM control solution was placed inside the dialysis tube (donor phase) containing 1-mL PBS at pH 7.4. The receptor compartment (phase) contained 49 mL of the same aqueous buffer used in the donor phase. A volume of 1-mL sample was taken from the receptor compartment at certain time intervals and then was replaced immediately with 1 mL of fresh phosphate buffer to maintain the same volume and to maintain sink conditions. The drug concentration in the withdrawn samples was measured using the microbiological assays described earlier.

#### 2.5.10. Azithromycin Penetration Study

Excised whole eyeballs of sheep were prepared freshly and stored at 4 °C within 1 h of slaughtering. A small transverse incision was made about 5 mm of surrounding sclera tissue from the excised eyeballs of sheep, and subsequently, the cornea was carefully cut out and washed with PBS (pH 7.4) to eliminate any adhering pigments. A certain volume of PBS with AZM films and AZM control solution was placed in the donor compartment at 37 °C, and then, the cornea was placed firmly on the donor so that the endothelial side was immersed in PBS at the receptor compartment. The donor compartment was at the top side of the receptor. One-milliliter samples were taken from the receptor compartment at specified time intervals and then replaced with the same volume of fresh medium for maintaining sink conditions [20,24]. The amount of AZM penetrated through the cornea was measured using microbiological assays. The code of ethics approved for this project is IR.KUMS.REC.1396.568.

#### 2.5.11. Ocular Irritation Study on Rabbit Eyes

A sterile insert containing AZM-Eudragit^®^ L100 nanoparticles was inserted into one eye of the rabbit, while the other eye was treated with PBS as the control. For the irritation test, the rabbits were examined within 72 h. As per the scoring system of guidelines for ocular irritation testing, the discharge, irritation, and redness of the corneal and conjunctiva were graded on a scale from 0 to 4 based on the articles published [25,26].

#### 2.5.12. In Vivo Studies

In vivo studies were conducted on healthy rabbit eyes. Male New Zealand rabbits weighing 3 to 3.5 kg were utilized to determine the release of azithromycin in the eye. There were 18 animals in the experimental group separated into three groups: (a) AZM film-HEC-treated, (b) an AZM film-HPMC group, and (c) an AZM solution-treated group [26]. Ocular inserts (20 mg, 6-mm length, and 0.18-mm thickness) and azithromycin solution were introduced into one of the rabbit’s conjunctival sacs, while the other rabbit’s eye was treated with PBS as the control. A specified amount of sample was withdrawn at regular time intervals. In this method, at the time of sampling, 30 μL of sterile phosphate buffer was poured into the eye and, then, the tear collected through the paper disc. The paper discs were transferred directly into the culture medium, which was already prepared to measure the microbial drug with the antibacterial assay method. The animal study was reviewed by the appropriate ethics committee of Kermanshah University of Medical Sciences (approval number IR.KUMS.REC.1396.568, approval date 3 January 2018).

### 2.6. Statistical Analysis

Results of this study are reported as mean ± SD. Kruskal-Wallis and *t*-tests were utilized to measure the statistical significance of the results. Differences were considered significant where *p* < 0.05. All the tests were performed in triplicate.

## 3. Results and Discussion

### 3.1. Evaluation of Nanoparticles

#### 3.1.1. Optimization of AZM-EUD NPs

**Figure 2:**
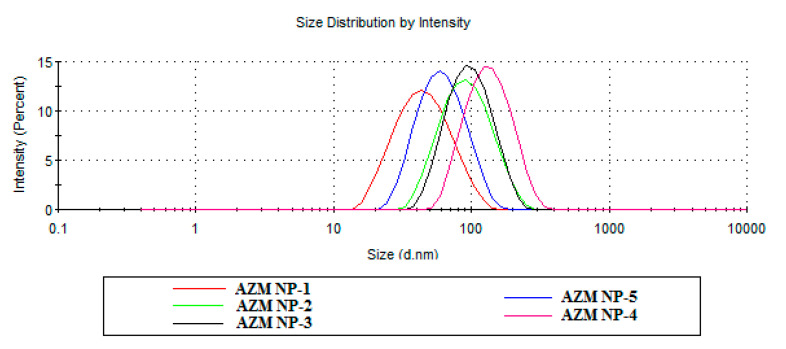
Particle size distribution of Eudragit^®^ L100 nanoparticles by Malvern Zetasizer.

In the optimization of AZM-EUD NPs, different factors such as size, zeta potential, and entrapment efficiency were considered by making different formulations, as listed in Table 1. This method was also successfully employed to produce Eudragit ^®^L100 NPs using PVA as a stabilizer [27]. The particle size distribution of different formulations of azithromycin Eudragit^®^ L100 nanoparticles was shown in Figure 2. AZM-EUD NPs displayed a homogeneous size distribution with polydispersity index (PDI) < 0.3, with mean particle sizes ranging between 38.58 ± 2.3 and 97.27 ± 4.3 nm for AZM-NP-1 and AZM-NP-3 formulations, respectively. When the amount of Eudragit^®^ L100 was kept constant, the amount of PVA increased the particle size, and the PDI of NPs decreased (Table 1). A reduction in PVA concentration failed to form a complete coverage of nanoparticles, which reduced the physical stability of the nanoparticles. This, in turn, can lead to coagulation of the particles, hence an increase in the size of the nanoparticles and PDI values. In Galindo-Rodriguez et al.‘s study, the lower level of PVA in the external aqueous phase led to the larger mean nanoparticle size [28]. Table 1 also shows that, at a constant concentration of PVA, an increase in the concentration of Eudragit resulted in an increase in the size of NPS. For instance, increasing Eudragit^®^ L100 concentrations from 2.5 to 10 mg/mL led to an increase in NP sizes from 38.58 ± 2.3 nm to 78.06 ± 2.3 nm (Table 1). The viscosity of the organic phase decreased as a result of a reduction in the polymer concentration, which can facilitate the distribution of the polymeric solution in the external phase. This may result in reducing the particle size and drug entrapment. On the other hand, the higher viscosity of the organic phase is contributed to the increase in the polymer-solvent and polymer-polymer interactions. Consequently, a more viscous organic phase led to a higher resistance against the diffusion of the polymer-solvent phase into the external aqueous phase, which caused an increase in the size of NPs [28,29].

The encapsulation efficiency of nanoparticles is shown in Table 1. The maximum amount of EE% (62.16 ± 0.07) was obtained for AZM NP-5. Additionally, an increase in the PVA concentration resulted in a higher viscosity of the external phase, which could prevent drug diffusion when NPs were being formed, which can lead to a higher %EE [28]. Increasing the viscosity of the organic phase (the phase Eudragit dissolved) resulted in good retention of the drug in the droplets, which can lead to high EE%. On the other hand, a reduction in the viscosity of the organic phase (where less Eudragit was used) could lead to moving the drug molecules to the droplet surfaces and being dispersed in the external phase (aqueous phase), leading to low EE% [29,30].

Pure anionic polymer nanoparticles caused negative zeta potential values, which originated from the carboxyl groups on the Eudragit L100 chain. One of the most important factors that affects zeta potential may be the existing of residual PVA on the surface of the nanoparticles. At the constant amount of Eudragit^®^ L100, increasing the PVA concentrations resulted in a reduction in negative zeta potential (i.e., zeta potential approaching zero). AZM-NP-3 showed the maximum amount of negative zeta potential, and AZM-NP-5 had the least negative zeta potential (Table 1). This happens due to the coating of the emulsifier (PVA), thus covering the possible charged groups on the particles [31].

It was observed that all the NPs have an acceptable particle size and PDI, but as AZM NP-5 showed the highest %EE, therefore, this formulation was selected for further investigation to make an ocular insert. Inserts of AZM were obtained by the addition of HPMC or HEC to AZM NP-5 nanoparticle solution (Eudragit^®^ (1% *w*/*v*), PVA (1% *w*/*v*), and drug:polymer (10% *w*/*w*), as shown in Table 2.

#### 3.1.2. FTIR Analysis

**Figure 3:**
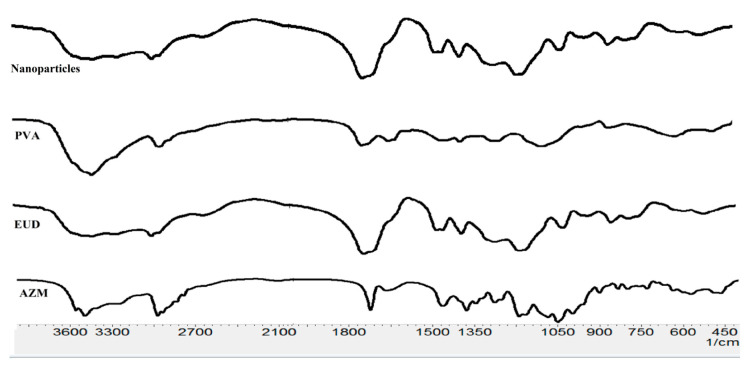
Fourier-transform infrared (FTIR) spectra of AZM, Eudragit^®^ L100, PVA, and AZM- Eudragit^®^ (EUD) nanoparticles (NPs).

The azithromycin spectrum showed a peak at 3490 cm^−1^ corresponding to O-H stretching vibration. The peak at 1720 cm^−1^ was assigned to C=O stretching vibrations. The peak detected at 1377 cm^−1^ is characteristic of -C-N stretching vibration. Peaks at 1453 and 1183 cm^−1^ corresponded to CH -O and C-O-C stretching vibrations. The IR of Eudragit^®^ L100 demonstrated some characteristic peaks at 3240 cm^−1^ (OH groups vibrations), 1385 cm^−1^, 1473.62 cm^−1^, and 2989.66 cm^−1^ (CHX vibrations); 1180 cm^−1^ and 1265 cm^−1^ (ester vibrations); and 1735 cm^−1^ (esterified carboxyl groups vibrations). In the FTIR spectrum of nanoparticles, the ester vibrations group of Eudragit^®^ L100 was found at 1265 and 1179 cm^−1^ (Figure 3). FTIR spectra of Eudragit^®^ L100 nanoparticles showed all characteristics bands for the polymer without any extra peak or shift in the bands. This ruled out any chemical interaction between the drug and polymer.

#### 3.1.3. Cytotoxicity Study

**Figure 4:**
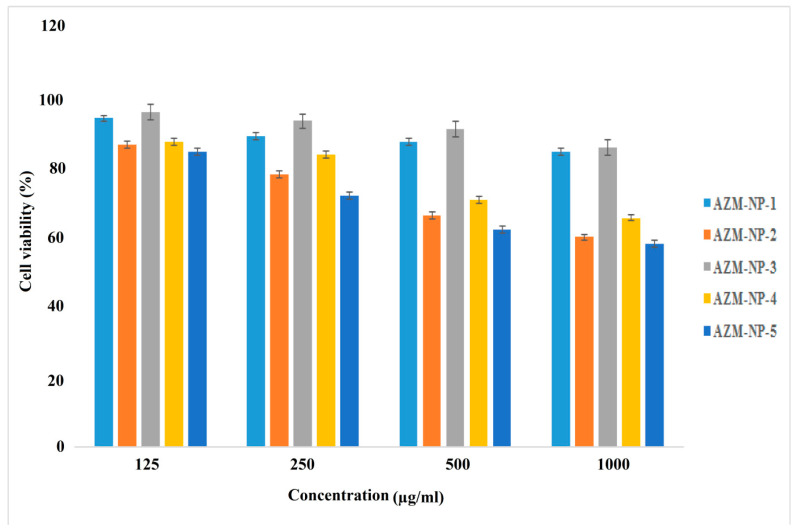
Cell viability of L929 (mouse fibroblast) cells (ATCC) after treatment with Eudragit^®^ L100 nanoparticles for different concentrations of nanoparticles: 125, 250, 500, and 1000 µL/mL. Cell viability measured by (3-(4,5-dimethylthiazol-2-yl)-2,5 diphenyl tetrazolium bromide) (MTT) assay. Data are expressed as the mean ± SD of three separate experiments (mean ± SD, *n* = 6).

The safety of different formulations of Eudragit^®^ L100 nanoparticles should be examined before applications for ocular delivery. In this study, all the prepared formulations were evaluated for the cytotoxicity test on L929 (mouse fibroblast) cells. As shown in Figure 4, the cell viability percentage after 48 h of incubation was related to the Eudragit^®^ L100 and PVA concentrations in the formulation. At constant concentrations of Eudragit^®^ L100, the cell viability percentage could decrease by increasing the PVA concentrations and at constant concentrations of PVA; increasing the concentration of Eudragit^®^ L100 in the formulation leads to cell viability reduction. For example, in 125-µg/mL concentration of formulations, the mean cell viability percentage ranged between 93.77 ± 1.32 for AZM-NP-5 and 84.21 ± 1.71 for AZM-NP-1. On the other hand, the cell viability percentage reduced for the formulation with higher NPs concentration (125–1000 µg/mL). Similar results were also reported when L929 (mouse fibroblast) cells were utilized for cytotoxicity study on PLGA nanoparticles as ocular carriers [18]. Finally, based on the results, all of the investigated AZM-loaded Eudragit^®^ L100 nanoparticles exhibited low cell cytotoxicity and were safe to be used as an ocular drug delivery system, but only AZM-NP-5 was selected to make an ocular insert as described earlier.

### 3.2. Characterization of Films Containing AZM-Loaded Eudragit^®^ L100 Nanoparticles

**Figure 5:**
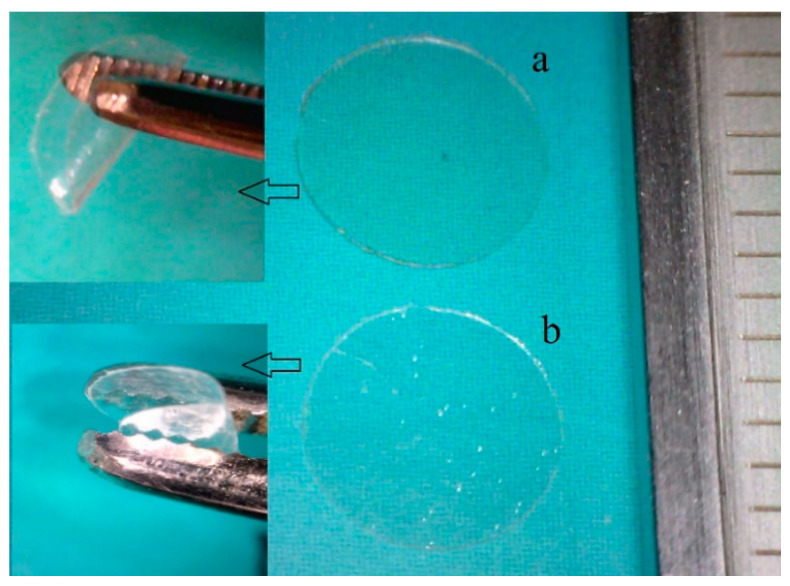
Ocular inserts with Eudragit^®^ L100 nanoparticles for azithromycin drug delivery. (**a**) AZM film-HPMC and (**b**) AZM film-HEC. Note: Scale: 1 mm.

The films containing AZM-Eudragit^®^ L100 nanoparticles were evaluated based on the physiochemical properties like thickness, folding endurance, and percentage of moisture absorption and loss, as illustrated in Table 3. Films containing NPs were colorless, transparent, self-standing, easily handled, flexible, and homogenous, with a smooth surface (Figure 5). The thicknesses of inserts were found to be 0.156 ± 0.008 mm for AZM-HEC and 0.164 ± 0.005 mm for AZM-HPMC, which were thin enough to be inserted into an ocular cul-de-sac without any eye irritation. The reported folding endurance for the prepared inserts was more than 200 (Table 3), which confirmed that they have enough flexibility and can easily get fixed in the conjunctival sac and can be considered satisfactory. The percentage of moisture uptake of inserts was found to be between 3.9% ± 0.4% to 5% ± 0.7%. Although, AZM-HEC had the highest amount of moisture loss (1.5% ± 0.3%), and AZM-HPMC showed the minimum amount of moisture loss (1.4% ± 0.2%) (Table 3), their difference was not significant. The results indicated that these ocular inserts have appropriate physical stability at high humid and dry conditions.

Swelling percentage plays a crucial role in the drug release profile from the polymeric matrix. Swelling of the inserts is affected by several factors like ionic strength, the concentration of the polymer, and the presence of water. Various types of hydrophilic polymers with different structures may have a different swelling percentage, and this is because they have specific abilities for the prevention of movement of water molecules through the matrix network [32]. The amount of swelling in AZM film-HEC was higher than AZM film-HPMC (Table 3). Water penetration into HPMC has been demonstrated to occur by two mechanisms, including capillarity through available pores, besides a penetration through the thick gel layer after sealing of the pores [33]. The elasticity and strength of the prepared inserts are related to the parameters like elongation at the break and tensile strength. A suitable bioadhesive ocular insert should have a relatively moderate tensile strength and elongation at the break. The tensile strengths of the inserts were found to be 22.6 ± 5.2 and 26.7 ± 3.6 MPa and elongations at the break (%) were 4.4% ± 0.3% and 5.1% ± 0.3% for HPMC film and HEC film, respectively (Table 3). HEC films showed higher tensile strengths and elongations at the break (%) than HPMC film during the initial study. The pH surface of AZM ocular inserts were found to be between 6.66 ± 0.05 and 6.83 ± 0.055, a range that is much closer to the ocular pH (6–7.6) (Table 2). This indicates that these inserts can be safely applied to any ocular tissue without causing any irritation.

#### 3.2.1. Morphological Characterization

**Figure 6:**
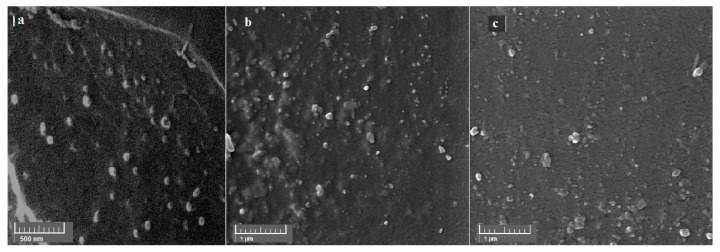
SEM images of (**a**) Eudragit^®^ L100 nanoparticles, (**b**) AZM film-HPMC, and (**c**) AZM film-HEC.

Morphological characterizations of the inserts using scanning electron microscopy (Figure 6) indicated a uniform distribution of AZM-EUD nanoparticles with rounded and some oblate shapes in the films. Mean particle size was like the mean diameter of nanoparticles obtained by the zetasizer.

The prepared films were introduced as the smooth and compact structures without any cracks and pores in the film matrix, and it is interesting to note that this uniformity in the film matrix was preserved after the addition of AZM-EUD nanoparticles into the films.

#### 3.2.2. Microbiological Assay Test

The quantification analysis of antibacterial agents like macrolides is commonly done by microbiological assays [34]. Different pharmacopoeias and many publications recommended the microbiological assays as a method to quantify antibiotics in the formulation [35,36]. The experimental assay used in this method has been performed by the Japanese Ministry of Health and Welfare Organization [37]. In most assays, the paper disc technique was introduced for the measurement of antibiotic effectiveness in drug delivery systems. Previous research has demonstrated that the sensitivity of the microbiological assay for *Bacillus subtilis* in the case of azithromycin incorporated into liposomes was 0.00390 mg/L [38]. Additionally, for the quantitative determination of azithromycin in ocular solution, the sensitivity of the microbiological assay was about 50 μg/mL [39]. In another study, Breier et al. indicated that determination of the azithromycin concentration in pharmaceutical dosage forms was carried out by the cylinder–plate method, and *Micrococcus luteus* was utilized as the test organism, and the calibration curve for azithromycin indicated good linearity in the concentrations range from 0.1–0.4 μg/mL [40]. For the first time, the authors of the current research employed the disc technique to quantify the azithromycin microbial assay through the direct collection of tears from the eye and transfer the disc to the plate that was described earlier in this manuscript.

**Figure 7:**
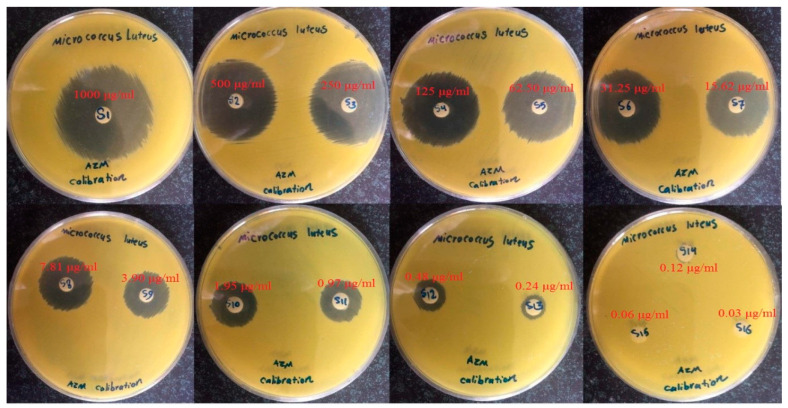
Zone of growth inhibition by *Micrococcus luteus* in the analysis of azithromycin.

In the present manuscript, the quantification of the released AZM in the tear fluid from different film formulations during the in vivo, in vitro, and ex-vivo trans-corneal permeability studies were performed by the paper disc method, which was described earlier. The lower detection limit of AZM with this method was 0.122 µg/mL. The integrity of the drug eluted from the Eudragit^®^ L100 nanoparticles was confirmed by the microbiological assay results. The growth inhibition zone diameter of the azithromycin standards was presented in Figure 7. For the standard AZM solutions, a linear correlation between the log10 of the drug concentrations and growth inhibition zone diameter was observed. The representative linear equation for *Micrococcus luteus* counts in the analysis AZM standards was y = 11.032x + 23.38. The calculated correlation coefficient (R^2^) was 0.9869, indicating good linearity. The CV% calculated for each concentration in the calibration curve was between 0.14% to 0.81%. To assess the accuracy and precision of the microbial assay, three samples of AZM with the concentration of 125, 62.5, and 31.25 µg /mL in three replicates were analyzed using *Micrococcus luteus* in the same day and on three different days. The results showed that intraday precision (CV%) was in the range of 0.54–0.81, and accuracy (%) was 95.07–117.78. Interday precision (CV%) was in the range of 0.25–0.95, and accuracy (%) were found to be in the range of 97.30–102.29. All these data indicate the suitability of the method to quantify azithromycin.

#### 3.2.3. Sterility and In Vitro Antimicrobial Efficacy Test

A sterility test was carried out to ensure that the prepared inserts were sterile. The results of this test demonstrated that there was no significant evidence of any microbial growth in the negative control test tubes and in the tubes containing ocular inserts. This indicates that all the tested inserts were sterile.

**Figure 8:**
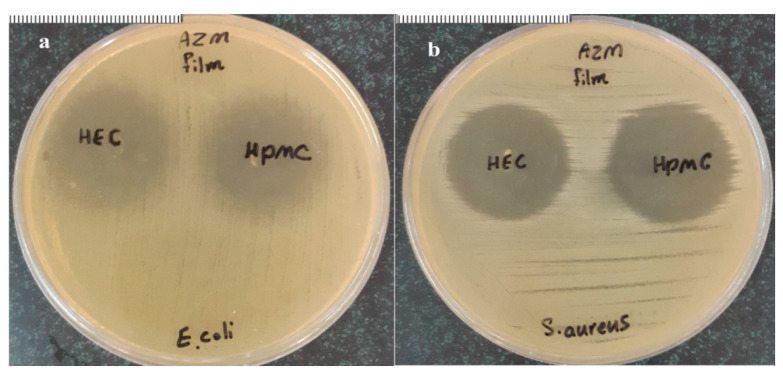
The areas of inhibited growth of (**a**) *Escherichia coli* and (**b**) *Staphylococcus aureus* around AZM film-HPMC and AZM film-HEC. Note: Scale: 1 mm.

The antimicrobial effectiveness of the prepared ocular inserts was evaluated by observing the inhibition zones of *E. coli* and *S. aureus* during 24 h in bacteria culture. Clear inhibitory zones were indicated around all the AZM inserts with both *E. coli* and *S. aureus* strains (Figure 8). Previous studies suggested that azithromycin-loaded polymeric nanoparticles were much more effective than the drug alone against *E. coli*, *Haemophilus* influenzae, *S. aureus* and *Staphylococcus pneumonia*. So, the clinical effectiveness of azithromycin might be enhanced if its polymeric nanoparticles are incorporated in the ocular dosage forms [15].

#### 3.2.4. In Vitro Studies

**Figure 9:**
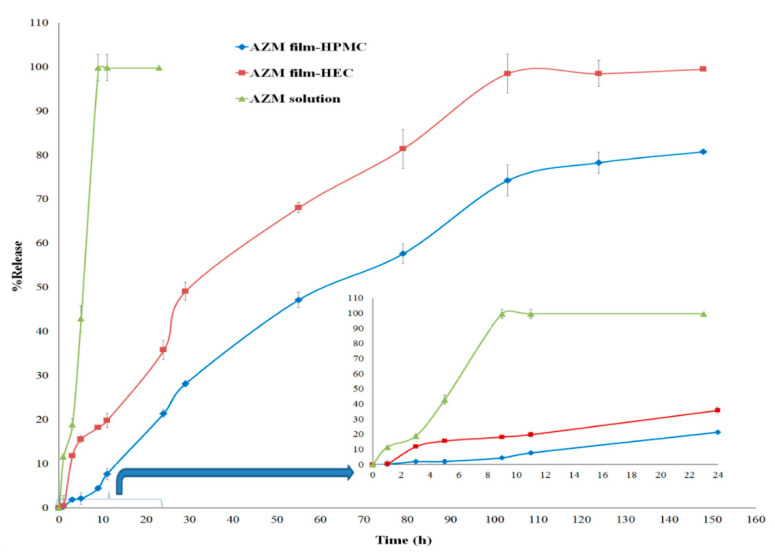
In vitro cumulative AZM release behavior of azithromycin from AZM film-HPMC, AZM film-HEC, and AZM solution. Release assay was performed in phosphate-buffered solution (PBS) solution, pH: 7.4, at 37 °C with agitation (mean ± SD, *n* = 6).

The results of the AZM release in phosphate-buffered solution (pH 7.4) are given in Figure 9. Both films containing Eudragit^®^ L-100 nanoparticles indicated a biphasic release pattern, including an initial burst release and then sustained release patterns. It has been shown that clarithromycin-loaded Eudragit^®^ L100 microspheres release the drug via first-order kinetics [41]. Another study carried out by Cetin et al. on Eudragit^®^ L100-PLGA nanoparticles showed a more sustained and controlled drug release (56–81% of the drug released over a period of 72 h) [42]. Our previous study on ketorolac tromethamine-loaded Eudragit^®^ L100 nanoparticles in the polymeric matrix showed that ketorolac tromethamine had an initial burst release from the hydrophilic polymeric matrix, followed by a slow release pattern in the second phase [27].

In the present study, azithromycin-loaded Eudragit^®^ L100 nanoparticles were prepared and reported that a controlled drug release of about 80.75–99.48% was obtained during six days (Figure 9). The initial mild burst could be due to the presence of free drug adsorbed onto the surfaces of the NPs or the presence of the free drug in the film. Nearly 20% of AZM was released from the HEC film during the first 10 h. This may have an effect on providing a higher dose of antibiotic at the beginning of ocular drug therapy [43]. During the next 24 h, 21.4% and 35.78% of the AZM were released from HPMC film and HEC film, respectively. After six days, almost all the drug was released from the inserts. The slow release pattern could be due to the adsorbed hydrophilic polymers to the surface of NPs, which could create an extra barrier to drug release and, hence, prolong the release of the drug [44]. The drug release rate in the HPMC film was relatively slower than that of the HEC film (Figure 9), which could be due to the lower swelling ratio of this insert and, hence, reduced the diffusion of the AZM from the film (Table 3). The azithromycin release data for each formulation were fitted to four kinetic models, including zero-order, Higuchi, first-order, and Peppas. The value of R^2^ related to each kinetic model was compared. The combination of polymer swelling, erosion, and diffusion through the hydrated matrix affected the drug release. The kinetic results showed that first-order was the appropriate fitting model for the HEC film, while the drug release from HPMC film followed zero-order, as shown in Table 4.

#### 3.2.5. Ex-Vivo Trans-Corneal Permeability Study

**Figure 10:**
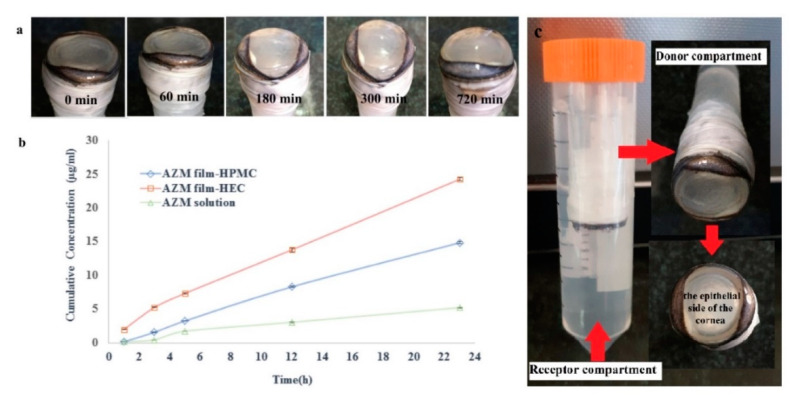
Ex vivo evaluation of AZM films. (**a**) Images of sheep eyes during experiment. (**b**) Azithromycin penetration profile from AZM film-HPMC, AZM film-HEC, and AZM solution. (**c**) The donor and the receptor compartment for penetration studies. Penetration assay was performed in PBS solution, pH: 7.4, at 37 °C with agitation (mean ± SD, *n* = 3).

According to United State Pharmacopoeia (USP) and The International Pharmacopoeia, the particle size for ocular administration must not be more than 10 μm. All the nanoparticles manufactured and incorporated in the ocular inserts demonstrated particle sizes between 38.58 ± 2.3 nm to 97.27 ± 4.3 nm, which could be very appropriate for ocular administration. The size of the nanoparticles is one of the most significant factors in defining their therapeutic efficacy, because the main purpose of ocular drug carrier is to enhance the performance of the formulation. Results of the cumulative corneal permeation of AZM-loaded Eudragit^®^ NPs from AZM-HEC, AZM-HPMC, and the pure drug solution (the same concentration of AZM in solution) are shown in Figure 10. The influence of different factors such as the lipid/water partition coefficient, chemical nature of the substance, size, and degree of ionization on the permeation of the drug to the corneal barrier is undeniable [45]. For a drug to penetrate to the cornea, the drug must be dissolved in lipophilic layers of the cornea, including the epithelium, hydrophilic layers like stroma, and less lipophilic layers than the epithelium, including endothelium. Many low molecular weight antibiotics have a limited capacity to overcome the corneal epithelium layers with different structural features, as they need to go through the paracellular route, where the transport of any chemicals is extremely limited by the presence of tight junctions [45]. The results of this study indicated that there was a remarkable difference between the cumulative permeation of the drug from the insert containing azithromycin-loaded Eudragit^®^ L100 NPs in comparison to the AZM solution. At 24 h, 24.28 ± 0.23 μg/mL, 14.9 ± 0.31 μg/mL, and 5.28 ± 0.13 μg/mL of AZM transported cumulatively across the cornea from HEC film, HPMC film, and AZM solution, respectively. This could be a result of the prolonged drug release from the insert. Moreover, the mucoadhesive properties of the film create an appropriate contact between the nanoparticles and corneal membrane, leading to a prolonged retention of the formulation at the site of administration, which can enhance the permeation of the drug. A similar finding has been reported for PLGA NPs by Qaddoumi et al. [46].

Furthermore, the hydrophobic character of the nanoparticles can increase the drug permeation. The study on Eudragit-based nanodispersion loaded with ketorolac tromethamine as the hydrophilic drug showed a significant improvement of drug permeation after 1.5 h due to the hydrophobicity of the nanocarrier that played a vital role [47]. The drug cumulative corneal permeation (μg) in the HEC film was almost higher than the HPMC film. This may be due to a lower swelling ratio of HPMC film compared to HEC film, which can reduce the diffusion of the AZM from the film and reduce the penetration of the NPs to the cornea. The apparent corneal permeability (Papp.) of azithromycin-loaded Eudragit^®^ L100 NPs and AZM solution was determined. The results indicated that the AZM permeability coefficient in the solution was 0.4 × 10^−6^ cm s^−1^ for the AZM solution, and the AZM film showed Papp of 2.77 × 10^−6^ and 1.89 × 10^−6^ cm s^−1^ for AZM-HEC and AZM-HPMC, which were 4.72 and 6.92 times higher than the AZM in solution. It was supposed that, during the in vivo study, azithromycin-loaded Eudragit^®^ L100 NPs in the film can promote ocular availability.

#### 3.2.6. Ocular Irritation Study on Rabbit Eyes

**Figure 11:**
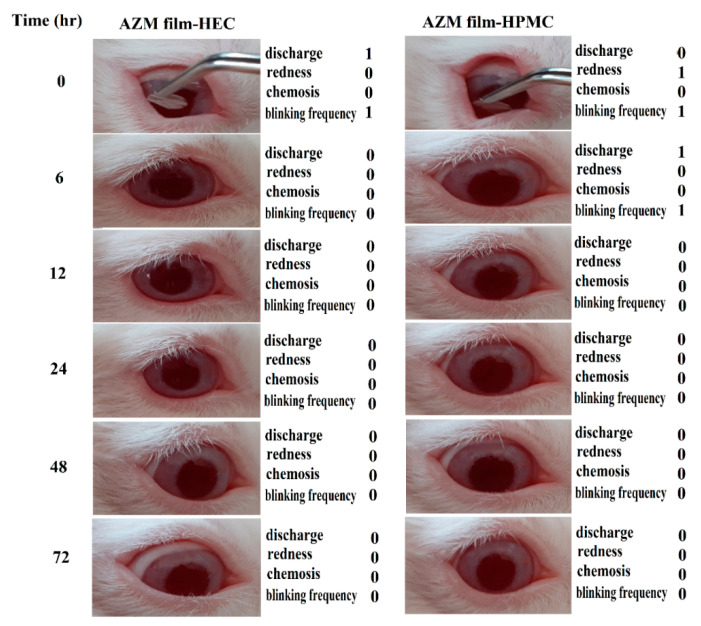
The appearance of rabbit eyes treated with a film containing azithromycin-loaded Eudragit^®^ L100 nanoparticles for ocular delivery acquired at 0 to 72 h.

Figure 11 represents the results obtained from the ocular irritation study of the prepared formulations. The right eye of the rabbit received AZM films, and left eye was treated with PBS as the control. The results showed that there were no symptoms of ocular irritation such as inflammation or swelling of the iris, conjunctival redness, chemosis, discharge, and blinking frequently after inserting the AZM film when considered up to 72 h. Grade 1 redness of conjunctiva and blinking were observed after the administration of inserts, which was reduced as time progressed. All the groups received eye irritation scores less than 1, indicating a high ocular tolerance of AZM films. The results of our study showed that the studied ocular inserts containing AZM-Eudragit NPs could be utilized as a safe ocular drug delivery system.

#### 3.2.7. In Vivo Studies

This study was performed on rabbit eyes by evaluation of the drug content in rabbit tears released from AZM films at different time intervals. In rabbits’ eyes, which were subjected for an in vivo study, any inflammation, irritation, discharge, and redness was not observed, which indicated that the selected polymers were appropriate polymers used in the formulations. The biodegradable AZM films were placed in the conjunctival sacs of rabbits as an ocular insert without requiring an invasive method. The amount of azithromycin released in tear fluid from AZM films was measured by the microbial assay method.

**Figure 12:**
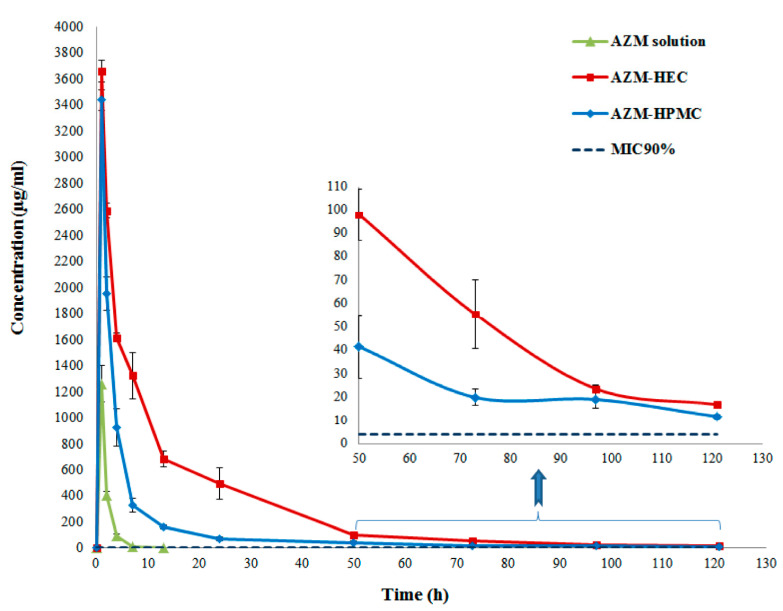
The concentration-time curves of AZM in tear samples for AZM film-HPMC, AZM film-HEC, and AZM solution (mean ± SD, *n* = 6). The amount of AZM solution (1% *w*/*v*; 1:1, ethanol:water) was two drops in this experiment, which as equivalent to the drug in inserts.

Figure 12 shows the concentration-time profiles of AZM in rabbit tears released from different AZM film formulations and the AZM in solution. The measured concentration of AZM solution after 1 h was 1257.4 ± 139.8 μg/mL, but this value dropped to 0.122 µg/mL after 13 h. A maximum concentration (C_max_) of 3435.9 ± 76.5 and 3659.4 ± 11.2 μg/mL were obtained for AZM-HPMC and AZM-HEC film, respectively, after one h inserting these films in the eye. The AZM films demonstrated an initial burst release followed by a gradual AZM release in tear fluid, exhibiting a prolonged release of the drug for 121 h. Area under the curve (AUC 0–121 h) of AZM in AZM-HEC, AZM-HPMC, and AZM solution was 36,912.0 ± 367.3, 14,846.7 ± 108.8, and 5211.0 ± 268.3 μg h/mL, respectively. These results revealed that the AUC of AZM-HEC and AZM-HPMC improved 7.48-fold and 2.84-fold, respectively, compared with the AZM solution. The mean residence time (MRT) of AZM-HEC and AZM-HPMC also enhanced significantly compared with the AZM solution due to the presence of Eudragit^®^ L100 nanoparticles in the mucoadhesive matrix. Our results suggest that Eudragit^®^ L100 nanoparticles in films may significantly enhance the pharmacokinetic behavior of the AZM solution.

Generally, the minimum inhibitory concentration (MIC_90_) of AZM for bacteria-infected conjunctivitis ranges between 0.05 and 4 μg/mL for the common Gram-positive and Gram-negative bacteria [48,49]. The results showed that AZM concentrations were above this MIC_90_ up to 121 h when these films were used. For example, the concentration of azithromycin in the tears at 121 h were 16.46 ± 0.79 and 23.28 ± 0.75 μg/mL for AZM-HEC and AZM-HPMC, which were 2.39-fold and 5.82-fold over the MIC, respectively. Although the highest drug levels in the tears were observed for the AZM solution in the initial hours, this concentration dropped below the MIC after 13 h. This indicates that the performance of the films containing AZM-Eudragit nanoparticles was much better than the AZM solution and can be used safely as a novel formulation to treat eye infections.

## 4. Conclusions

The current research involved the preparation, in vitro study, ex-vivo trans-corneal permeability study, and in vivo evaluations of mucoadhesive biodegradable inserts containing azithromycin-loaded Eudragit^®^ L100 nanoparticles that could be effective as an antibacterial topical ocular drug delivery carrier for the first time. The mucoadhesive properties of AZM films can stick the films to the surface of the cornea, which could prolong the ocular retention time and lead to the enhanced performance of inserts in the ocular tissues. In vitro studies demonstrated a controlled release of the drug during a long period (148 h). The MTT cell viability experiment was also carried out using L929 fibroblast cells, which showed that AZM films were not significantly cytotoxic. The in vivo study results exhibited that the incorporation of NPs in the mucoadhesive matrix could be considered as a good way to prolong the drug release and reduce the frequency of administration, leading to the enhanced patient compliance.

## Figures and Tables

**Table 1:** Preparative characteristics and physicochemical properties of the different formulations of azithromycin (AZM)-loaded Eudragit^®^ L100 nanoparticles (mean ± SD, *n* = 3).

Formulation	Eudragit (mg/mL)	PVA (mg/mL)	Drug: Eudragit (%W/W)	Particle Size (nm)	Polydispersity Index	Zeta Potential (mV)	EE * (%)
AZM-NP-1	2.5	10	10	38.5 ± 2.3	0.222 ± 0.003	−3.58 ± 0.90	32.14 ± 0.09
AZM-NP-2	5	10	10	53.4 ± 3.7	0.198 ± 0.005	−3.7 ± 2.30	46.30 ± 0.12
AZM-NP-3	10	2.5	10	97.2 ± 4.3	0.232 ± 0.009	−6.07 ± 1.02	52.29 ± 0.32
AZM-NP-4	10	5	10	81.7 ± 5.1	0.209 ± 0.003	−3.32 ± 1.60	55.06 ± 0.24
AZM-NP-5	10	10	10	78.0 ± 2.3	0.179 ± 0.007	−2.45 ± 0.69	62.16 ± 0.07

* Entrapment efficiency.

**Table 2:** Preparative characteristics of the ocular inserts of azithromycin. HEC: hydroxyethyl cellulose and HPMC: hydroxypropyl methylcellulose.

Formulation	Eudragit (mg/mL)	PVA (mg/mL)	Drug: Eudragit (%)	HEC (mg/mL)	HPMC (mg/mL)
AZM film—HEC	10	10	10	0.66	-
AZM film—HPMC	10	10	10	-	0.66

**Table 3:** Physicochemical parameters of the ocular inserts of azithromycin (mean ± SD, *n* = 3).

Formulation	Thickness (mm)	Folding Endurance	Moisture Absorption (%)	Moisture Loss (%)	Swelling (%)	Elongation at Break (%)	Tensile Strength (MPa)	pH (Mean ± SD)
AZM film-HPMC	0.164 ± 0.005	202.0 ± 6.6	3.9 ± 0.4	1.4 ± 0.2	207.5 ± 4.1	4.4 ± 0.3	22.6 ± 5.2	6.6 ± 0.1
AZM film-HEC	0.156 ± 0.008	267.6 ± 8.0	5.0 ± 0.7	1.5 ± 0.3	231.7 ± 0.6	5.1 ± 0.3	26.7 ± 3.6	6.8 ± 0.1

**Table 4:** The R-squared of the fitted kinetic models for different formulations.

Kinetic Models	HPMC Film/RSQ *	HEC Film/RSQ *
Zero	0.988	0.972
first	0.984	0.983
Higuchi	0.920	0.962
Peppas	0.985	0.948

* R squared.
